# Development and validation of a disease-specific quality of life scale for adult patients with Fabry disease in Japan

**DOI:** 10.1186/s41687-022-00525-z

**Published:** 2022-11-17

**Authors:** Yuta Koto, Wakana Yamashita, Yoko Lee, Nozomi Hadano, Chikara Kokubu, Norio Sakai

**Affiliations:** 1 Child Healthcare and Genetic Science Laboratory, Division of Health Science, Graduate School of Medicine, Osaka University, Osaka, Japan; 2 Faculty of Health Science, School of Nursing, Osaka Aoyama University, Osaka, Japan; 3 Department of Clinical Genomics, Saitama Medical University, Saitama, Japan

**Keywords:** Fabry disease, Quality of life, Surveys and questionnaires, Patient-reported outcome measures, Scale development and validation

## Abstract

**Background:**

Fabry disease is a rare X-linked lysosomal storage disorder. It is associated with physical distress and social challenges that may affect adults differently compared to pediatric patients. However, there is no disease-specific quality of life (QOL) scale that can provide a detailed assessment of QOL for adults with Fabry disease. Therefore, we aimed to determine the factor structure and assess the validity of a scale that was created to assess the QOL of adult patients with Fabry disease. This study was conducted in two phases. First, scale feasibility was confirmed through a questionnaire survey of nine patients. Second, a cross-sectional questionnaire survey of patients (aged ≥ 18 years) diagnosed with Fabry disease was conducted. Item development and refinement were conducted based on guidelines for scale development. Exploratory factor analysis was used to clarify the factor structure and confirm internal consistency. As a measure of QOL, construct validity was of the scale was verified based on its correlations with the Short Form-8 (SF-8) scale.

**Results:**

The newly created Adult Fabry Disease QOL (AFQOL) scale comprises 39 items that cover five factors: “neuropathic pain and abdominal symptoms,” “impact on work and school,” “relationship challenges,” “ophthalmologic and otolaryngologic symptoms,” and “cardiovascular and renal symptoms.” Cronbach’s alpha coefficient for all factors was above 0.8, and the AFQOL total scores were significantly correlated with the physical and mental components of the SF-8 (*r*s = − 0.508 and − 0.400, respectively).

**Conclusions:**

The AFQOL scale assesses physical symptoms and social difficulties experienced by adult patients with Fabry disease. A strength of the scale is its ability to assess the impact of work and relationships on patients. The scale can be useful in objectively assessing QOL for a group or for individual patients. Future research should explore further aspects of the scale’s validity and reliability.

**Supplementary Information:**

The online version contains supplementary material available at 10.1186/s41687-022-00525-z.

## Plain English summary

Adults with Fabry disease experience severe challenges, which adversely impact their quality of life (QOL). As it is a rare disease, non-patients lack awareness of the severity of its symptoms and the resultant social difficulties of the patients. Most instruments that measure QOL are not specific enough to address issues related to Fabry disease. Therefore, in this study, a measurement instrument known as the Adult Fabry QOL (AFQOL) scale was designed and validated. The 39-item scale covers five domains that are congruent with the symptoms of adult Fabry disease. It differs from other QOL scales as it also assesses the impact of work and personal relationships on patients’ QOL and symptoms that progress in adulthood. This study has important implications for healthcare providers who treat adult patients with Fabry disease, enabling them to have a fuller picture of the unique needs of this population.

**Supplementary Information:**

The online version contains supplementary material available at 10.1186/s41687-022-00525-z.

## Background

Fabry disease is a rare X-linked lysosomal storage disorder, caused by a deficiency of a lysosomal enzyme—alpha-galactosidase A—that leads to substrate accumulation throughout the body [1]. This accumulation in cells causes symptoms to appear throughout childhood and adolescence, leading to eventual multiple organ failure [1]. Hence, patients with Fabry disease have various symptoms such as neuropathic pain (shooting or burning pain with a low to severe intensity), abdominal symptoms, tinnitus, cardiac rhythm disturbances, renal failure, and stroke [1]. Fabry disease is classified into three phenotypes, the “classical” type, the “late-onset” type, and the “heterozygous female” type. Because the clinical manifestations of Fabry disease represent a wide spectrum, classification of these types might be inaccurate, but it is practical [2]. Patients with the classical type experience neuropathic pain and gastrointestinal symptoms from childhood, while those with the late-onset type vary in terms of age of onset and manifestations. The disease in heterozygous female patients ranges from being asymptomatic to the more severe phenotype [1].

Studies have estimated the prevalence of Fabry disease to range between 0.85 and 1.29 per 100,000 live births [3, 4]. The number of patients with Fabry disease in Japan was recently estimated at 1658 [5]. However, newborn screening results reported 14.17 per 100,000 live births, suggesting that the above results may be an underestimation [6]. For the classical type, the mean age of diagnosis is between 8 and 20 years, while for the late-onset and heterozygous female types, the mean age of diagnosis is approximately 40 years [5, 7].

Appropriate treatment of Fabry disease is important to prevent the progression of symptoms and organ damage. Currently, treatments that are available to patients are enzyme replacement therapy (ERT) and chaperone therapy [1]. ERT involves intravenous administration every two weeks, which is burdensome for patients and their families [1]. Chaperone therapy is an oral treatment, which is only given to individuals with specific genetic mutations [1]. Ancillary treatments include the use of drugs for pain and symptomatic treatment for circulatory and renal symptoms [1].

Patients with Fabry disease experience distress not only caused by systemic symptoms but also by the lifestyle restrictions associated with the treatment and the social consequences of living with a rare disease. Using the Wilson and Cleary model as a conceptual framework for understanding quality of life (QOL), it may be helpful to consider biological variables and the impact of symptoms and physical functioning when assessing the health related QOL of patients with chronic diseases [8]. A systematic review of the QOL of patients with Fabry disease showed that they had a lower QOL than the general population [9], and this has also been supported by more recent studies [10, 11]. Contrastingly, Arends et al. found that the change in QOL with ERT was small using general QOL scales [9]. Although general QOL scales are useful for comparing different populations, they are not sensitive enough to detect small changes in QOL, and a disease-specific QOL scale might be more sensitive to the effects of ERT on QOL [9]. At present, a QOL scale has been developed for pediatric patients with Fabry disease; it is available in multiple languages, including Japanese [12, 13]. However, because this scale is intended for pediatric patients and focuses on neuropathic pain and abdominal symptoms, it is difficult to extend its scope to adult patients who may be experiencing heart and kidney symptoms [2]. Although recent studies have suggested several patient-reported outcome measures for adult patients [14, 15], they only focus on some symptoms and severity and do not provide an overall assessment of the QOL of adult patients with Fabry disease.

As no comprehensive QOL scale exists for adult patients with Fabry disease, we developed a scale following the COnsensus-based Standards for the selection of health Measurement Instruments (COSMIN) guidelines [16]. A systematic review of qualitative studies of patients with lysosomal disease (including Fabry disease) undergoing ERT revealed a lack of reporting on the experiences of male patients with Fabry disease [17]. Furthermore, an investigation of the daily life experiences of adult patients, specifically men, identified items that would ensure a content-validated scale [18]. This study revealed that adult patients experienced difficulties related to diagnosis, treatment, social life, and family relationships, along with disease symptoms [18]. Consequently, we developed a tentative Fabry disease-specific QOL scale—the Adult Fabry Disease QOL scale (AFQOL). The purpose of this study is to clarify the item structure and internal consistency of this scale.

## Methods

### Study design and participants

This study was conducted in two phases. First, we used codes from a previous qualitative study [18] to develop the questions’ text. The codes represented each of the symptoms or difficulties experienced by the patients. The conversion from codes to questions was completed in discussion with physicians and nurses. Additionally, advice regarding the appropriateness of the linguistic expressions in the conversion was obtained from a patient. When preparing the items, we referred to Devellis’s scale development guidelines and took care to avoid multiple meanings, double negatives, and the use of ambiguous pronouns [19]. Then, 80 items were prepared and pretested to verify participants’ linguistic understanding of the questions and the answerability of the questions. The pretest was conducted with nine of the eleven participants from the aforementioned study; two changed outpatient hospitals between the qualitative research period and the pretest and therefore could not participate.

To conduct the pretest, the 80-item draft was sent to the participants. The questionnaire items were rated on a five-point scale, ranging from “always” to “never,” and a “not appropriate as a question” option was provided. The “no children” option was also provided for answering several items related to the participant’s children. Participants were told at the beginning of the questionnaire to select “not appropriate as a question” if they found the items difficult to understand, read, or answer. After tabulating the pretest results, we checked each item that was selected as “not appropriate as a question” to consider changing the wording of the text or removing the item altogether. When removing items, we carefully evaluated whether the differences were owing to the participant’s age, gender, or treatment experience, and not because of their lack of experience. For example, if an item related to a parent was selected as “not appropriate as a question” by an older patient, we concluded that this was because the parent had passed away, which was not a valid reason to exclude the scale item. The pretest was conducted between April and May 2021.

The second phase comprised a cross-sectional questionnaire survey of patients (aged 18 years or older) diagnosed with Fabry disease. Patients younger than 18 years and those without a confirmed diagnosis of Fabry disease were excluded.

The questionnaires were distributed to 200 members of the only two Fabry disease patient associations in Japan. All patients who met the inclusion criteria were confirmed beforehand with the associations—165 patients from one association and 35 from the other. Because Fabry disease is a rare disease and the number of patients is limited, this study did not specify a sample size by power of detection, but rather the maximum number of participants to whom the questionnaire could be distributed. The purpose and methods of the study and ethical considerations were explained in writing. The document also stated that participation was voluntary, that no one would be disadvantaged if they did not participate, that personal information would be protected, and that the collected data would be properly stored and later destroyed. All participants provided informed consent before taking part in the study. The questionnaires were anonymous and were collected by mail. The survey was conducted between November and December 2021.

### Instruments

The questionnaire consisted of three parts: (1) a background section on patients’ sex, age, diagnosis, and treatment; (2) a candidate questionnaire for the scale; and (3) the Short Form-8 (SF-8).

The candidate scale questionnaire consisted of 79 items, with one item excluded as a result of the pretest. The items all enquired about symptoms and social difficulties related to Fabry disease and responses were rated on a 5-point Likert-type scale (*always, often, sometimes, seldom,* and *never*): 4 = “always” and 0 = “never.” All scores were summed, and higher scores indicated a more severe condition. With the exception of questions relating to marriage and having children, all questions enquired about the previous month. Of the 79 items, 21 were reverse-scored (see Additional file 1: Table S1).

The SF-8 is a standard 8-item QOL scale covering eight domains and a physical and Mental Component Summary (PCS and MCS) that can be calculated [20, 21]. The SF-8 summary score is standardized with 50 as the mean; higher scores indicate a higher QOL. The SF-8 was used to assess the construct validity of the scale that was being developed.

### Analysis methods

The methods proposed by COSMIN were used to examine the reliability and validity of the scale [16]. The evaluation of the scale questions was based on Devellis’s scale development guidelines [19], including the calculation of item-total correlations and Cronbach’s alpha coefficient as well as exploratory factor analysis.

The measures used in this study were constructed based on the findings of a qualitative study [18]; medical accuracy in item wording was assessed by a physician specializing in Fabry disease. In addition, the items were checked by physicians and nurses to ensure that they adequately reflected the patients’ experiences from the preceding interviews [18]. Advice from a patient was also sought during this process. Linguistic comprehension, answerability of the scale’s questions, and face validity were assessed in the pretest. Items to which three or more of the participants did not respond in the pretest, or which were deemed inappropriate, were targeted for exclusion.

The first section of the main survey provided descriptive statistics. Regarding the interpretability of the response results, the distribution of scores was evaluated, and if the responses were concentrated on a particular option, the exclusion of such items was considered. As it was difficult to determine the threshold for exclusion, we checked the distribution of responses and considered exclusion after discussion. Next, to assess the internal consistency, item-to-item correlations and item-total correlations were calculated using Pearson’s correlation. Because the presence of symptoms and challenges on this scale results in higher scores, no response to a question was interpreted as the absence of symptoms or challenges and received a score of zero. Items with item-to-item correlations exceeding 0.8 were subject to exclusion. The decision to exclude either item was based on the results of the factor analysis that followed. Items with item-total correlations of less than 0.3 were also considered for exclusion [22].

An exploratory factor analysis was conducted to examine the factor structure of the scale. First, a factor analysis was conducted with varimax rotation using principal axis factoring, and a scree plot was used to assume the number of factors. The number of factors was then fixed and a promax rotation was performed using principal axis factoring. As this scale consists of a set of items that encompass a variety of symptoms related to the body and social life, it does not assume that responses to all items are normally distributed. Therefore, principal axis factoring was adopted as the estimation method in the factor analysis. Items with commonality less than 0.3 after factor extraction were considered for exclusion [22]. For item pairs for which the inter-item correlation was greater than 0.8, the one with the lower commonality after factor extraction was excluded.

Cronbach’s alpha coefficients were calculated to confirm the internal consistency of the subscales identified by the factor analysis. The extracted factors were named following discussions with the physicians and nurses to reflect the items included in the factors. A patient’s advice was also obtained during this process. To determine the construct validity for the measurements of QOL, Spearman’s correlation between each subscale and total score and the SF-8 were calculated. We believed that the negative correlation between AFQOL and SF-8 could indicate that the AFQOL is valid for measuring patients’ QOL. Statistical analyses were performed in SPSS version 26.

## Results

### Pretest results

In the pretest, nine participants reviewed all the questions and either selected from various options or judged the item “not appropriate as a question.” Of the 80 questions, one or more participants viewed 27 of them as “Not appropriate as a question.” Two questions were considered inappropriate by at least three of the participants: QIV-10, “Do you feel that there is insufficient education about genetic diseases in school?” and QVI-6, “Do you have trouble socializing with relatives due to Fabry disease?” After discussion by a team that included physicians and nurses, QIV-10 was removed prior to the main survey because it was not directly related to participants’ QOL. The other items that were viewed as “Not appropriate as a question” were retained in the main survey since it was thought that participants’ age or marital status influenced their response to these items.

### Descriptive characteristics

We obtained 83 completed questionnaires (41.5%; 57 women and 26 men). As all respondents answered most of the questions, all collected questionnaires were considered valid responses.

Participants’ mean age was 52.2 (standard deviation [SD] = 14.90) years. The mean age at diagnosis was 37.9 (SD = 15.99) years. Regarding the method of diagnosis, 63.2% of the women were diagnosed by family history and 52.6% by genetic testing. In contrast, 46.2% of the men were diagnosed by enzyme activity test and 30.8% by family history. Regarding previous treatment experience, 88.0% were on ERT, 36.1% were using analgesics, and 13.3% were using chaperone therapy. Cardiac medications were used by 26.5%, renal medications by 13.3%, and hearing loss medications by 7.2%. When asked to rate their degree of pain on a scale of 0 (*not at all*) to 9 (*most painful*), the mean score was 1.5 (SD = 1.67), the median score was 1, and the range was 0 to 7.

### Examination of scale items

**Fig. 1:**
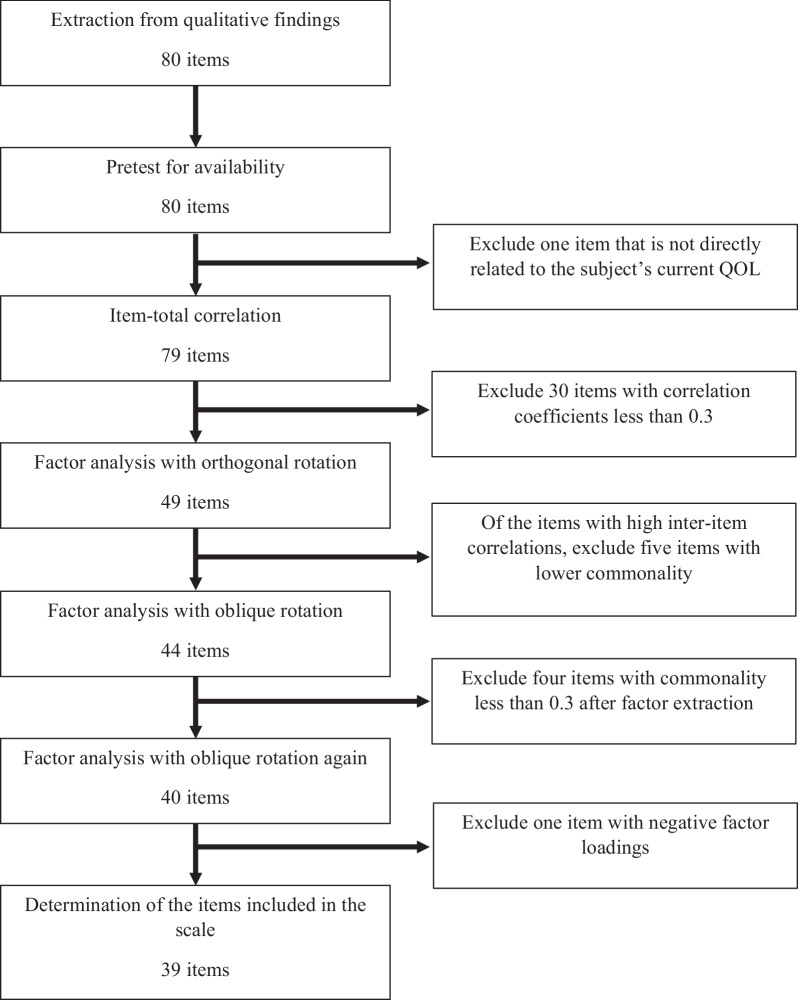
Item selection process

The number of items excluded by item screening is shown in Fig. 1 (see Additional file 1: Table S1 for details on the item selection process). The distribution of responses was checked, and no item had more than an 80% concentration on one option (see Additional file 1: Table S2). In Section V, the instructions explained that participants who were not working or not going to school should not respond. There were more than 30% non-responses to the items in that section, and most non-responders skipped the section entirely. Therefore, we decided to further examine the Section V items for scale item selection.

Nine pairs of item-item correlations were greater than 0.8 (Table 1). The decision to exclude either of these pairs was based on the results of a factor analysis. In addition, 29 items were excluded from the scale because they were less than 0.3 on the first item-total correlation. Two pairs with high inter-item correlations were excluded at this stage. After excluding 29 items, another question was excluded following the item-total correlation that was conducted again (Additional file 1: Table S1).

**Table 1:** High item-item correlation pairs (Pearson’s correlations; *N* = 83)

Question text		*r*
I-1: Do your toes begin to hurt when the ambient temperature or your body temperature rises?	I-2: Do your fingers begin to hurt when the ambient temperature or your body temperature rises?	0.911
I-3: Do you feel pain in your elbows when the ambient temperature or your body temperature rises?	I-4: Do you feel pain in your knees when the ambient temperature or your body temperature rises?	0.809
I-1: Do your toes begin to hurt when the ambient temperature or your body temperature rises?	I-5: Are you unable to exercise due to pain?	0.801
I-2: Do your fingers begin to hurt when the ambient temperature or your body temperature rises?	I-6: Are you unable to perform your daily activities due to pain?	0.803
I-5: Are you unable to exercise due to pain?	I-6: Are you unable to perform your daily activities due to pain?	0.815
I-7: Do your hands begin to hurt on cold days?	I-8: Do your feet begin to hurt on cold days?	0.865
I-10: Do you sweat on hot days?	I-11: Do you sweat while exercising?	0.952
V-1: Do you feel that the Fabry disease symptoms impact your work or schooling?	V-3: Do you feel that working outside is difficult?	0.800
VI-9: Do you feel sorry that your child(ren) is/are suffering from the symptoms of Fabry disease?	VI-10: Do you feel sorry that your child(ren) is/are suffering because they have Fabry disease?	0.882

**Fig. 2:**
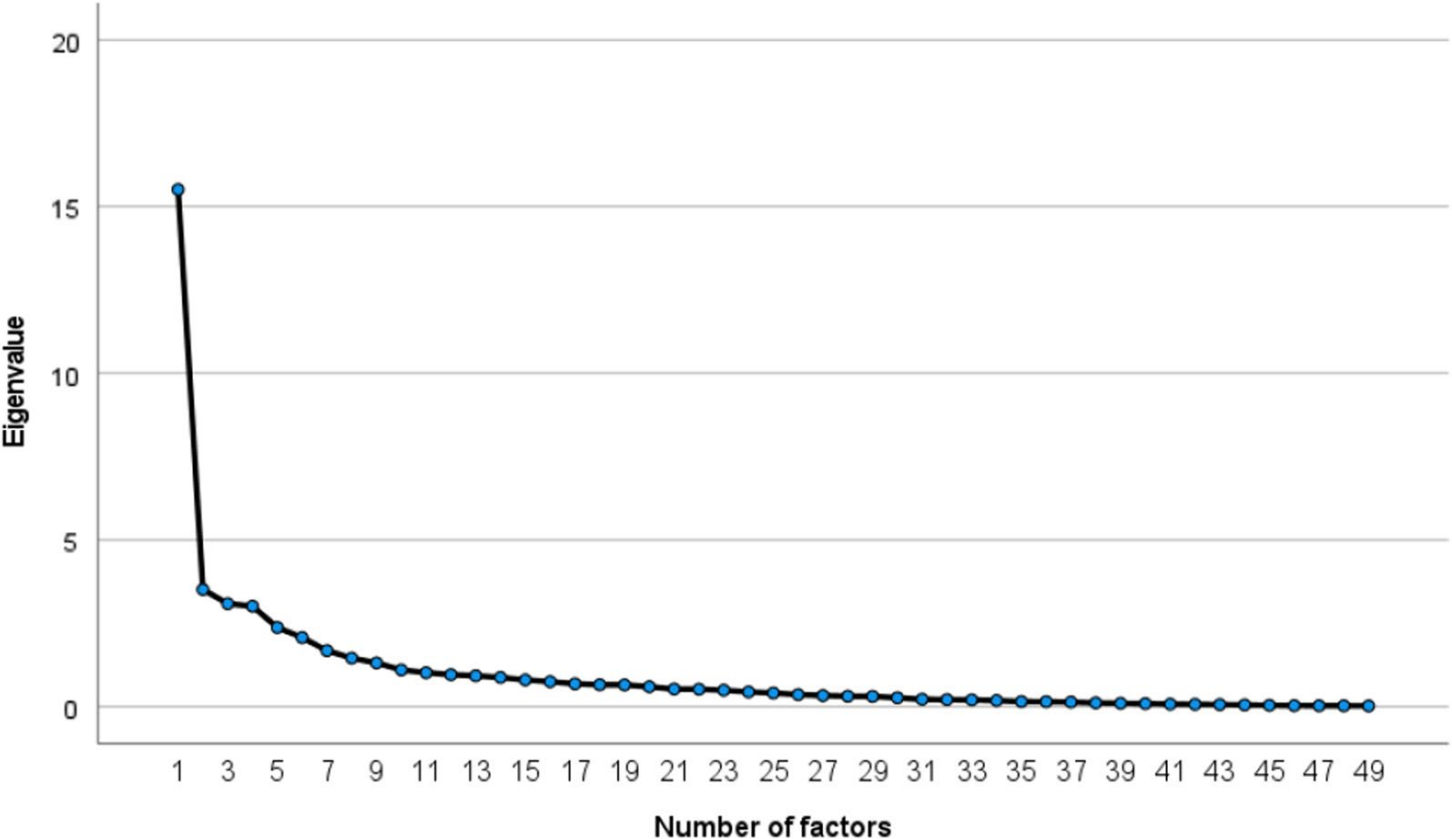
Scree plot generated from exploratory factor analysis

An exploratory factor analysis was conducted on the remaining 49 items to obtain the scree plot, as shown in Fig. 2. There could be four or five factors. However, when there were four factors, we determined that a medical interpretation would be difficult because the items related to neuropathic pain were divided into three factors, and neuropathic pain is a characteristic symptom of Fabry’s disease. Therefore, it was assumed that there were five factors, and a factor analysis with oblique rotation with the number of factors fixed at five was performed. At this stage, for the seven pairs with high inter-item correlations (two pairs were excluded due to low item-total correlations), the items with the lower commonality value were excluded. In the factor analysis, four items with a commonality value below 0.3 after factor extraction were excluded. Factor analysis was conducted again with the remaining 40 items, and one item (QII-4) showed negative factor loadings in the converged results. Therefore, this item was considered inappropriate for the scale and was excluded. Table 2 shows the factor loadings resulting from the factor analysis with 39 items. With the number of items finalized, the total AFQOL score went from a minimum score of 0 to a maximum score of 156.

**Table 2:** Factor loadings for factor analysis with oblique rotation

		Factor				
Item	Question text	1	2	3	4	5
I-1	Do your toes begin to hurt when the ambient temperature or your body temperature rises?	**0.799**	− 0.125	− 0.004	− 0.047	0.052
I-6	Are you unable to perform your daily activities due to pain?	**0.766**	0.036	0.073	− 0.128	0.030
I-4	Do you feel pain in your knees when the ambient temperature or your body temperature rises?	**0.729**	− 0.059	− 0.135	− 0.190	0.290
I-7	Do your hands begin to hurt on cold days?	**0.664**	− 0.027	0.027	0.344	− 0.160
I-13	Do you experience any pain when your body temperature rises in the bath?	**0.658**	− 0.026	0.054	− 0.029	0.096
I-12	Are you immobile on hot days?	**0.646**	0.191	− 0.031	0.101	0.014
I-22	Do you have diarrhea?	**0.585**	0.192	0.010	0.028	− 0.194
I-9	Do your hands begin to hurt while using tap water?	**0.448**	− 0.018	0.052	0.376	− 0.056
I-24	Do you experience abdominal pain?	**0.405**	0.059	0.149	0.359	− 0.179
I-25	Do you feel nauseous?	**0.396**	0.006	0.197	0.270	0.029
V-8	Do you feel bitter because of prejudice toward Fabry disease at your workplace or school?	− 0.129	**0.807**	0.199	0.032	− 0.099
V-9	Do people at your workplace or school ever make snide remarks about your Fabry disease symptoms?	− 0.134	**0.780**	0.194	0.054	− 0.062
V-2	Does your Fabry disease treatment impact your work or schooling?	0.013	**0.753**	− 0.063	0.015	0.050
V-4	Do you feel that your hearing difficulty affects work or schooling?	0.079	**0.729**	− 0.126	0.195	− 0.057
V-3	Do you feel that working outside is difficult?	0.354	**0.698**	− 0.033	− 0.180	0.034
V-7	Do you get scolded by those around you because you become irritable due to pain from Fabry disease?	0.119	**0.574**	0.133	− 0.097	− 0.053
IV-5	Do you feel hurt because the people around you do n’t understand you?	− 0.092	− 0.011	**0.741**	0.143	0.065
IV-6	Do you feel that you can’t properly explain Fabry disease symptoms to the people around you?	− 0.007	0.082	**0.635**	0.102	− 0.040
III-1	Do you worry about your Fabry disease?	0.218	0.086	**0.603**	− 0.314	− 0.043
IV-2	Do you feel that playing or doing activities with friends is difficult because of the Fabry disease?	0.281	− 0.046	**0.517**	0.096	0.049
III-4	Do you feel anxious about your future because of the Fabry disease?	0.445	− 0.031	**0.462**	− 0.329	0.002
VI-6	Do you have trouble socializing with relatives due to the Fabry disease?	− 0.212	0.348	**0.462**	− 0.045	0.045
I-33	Do you consider yourself mentally weak?	0.200	− 0.014	**0.458**	0.188	− 0.072
I-31	Do you get dizzy?	0.009	− 0.137	**0.407**	0.403	0.134
I-32	Do you feel you tire easily?	0.296	0.013	**0.393**	0.213	− 0.001
I-21	Do you have trouble socializing due to Fabry disease symptoms?	0.334	0.074	**0.379**	0.077	0.131
II-6	Are you concerned about side effects from your current treatment?	− 0.251	0.245	**0.313**	0.197	0.267
I-26	Do you feel that lights are too bright?	− 0.076	− 0.106	0.093	**0.820**	− 0.022
I-27	Do you have difficulty seeing at night?	− 0.132	− 0.022	0.185	**0.766**	− 0.066
I-29	Do you feel that it is difficult to have conversations because of the ringing in your ears?	0.099	0.416	− 0.261	**0.518**	0.123
I-28	Do you hear ringing in your ears?	0.141	0.192	− 0.198	**0.480**	0.100
I-30	Do you experience sudden, intense ringing in your ears?	0.189	0.280	− 0.238	**0.408**	0.139
I-15	Do you feel pain in your chest or difficulty breathing while walking?	0.034	− 0.142	0.023	0.103	**0.861**
I-14	Do you experience palpitations?	− 0.087	− 0.060	− 0.065	0.288	**0.778**
I-17	Do you feel anxious that your heart symptoms will get worse?	0.143	− 0.008	− 0.067	− 0.256	**0.724**
I-16	Do you feel pain in your chest or difficulty breathing while climbing stairs?	0.155	− 0.170	0.029	0.160	**0.561**
II-5	Are you anxious about the effectiveness of your current treatment?	− 0.249	0.319	0.185	− 0.053	**0.512**
I-20	Do you feel anxious that your kidney symptoms will get worse?	0.059	0.234	0.163	− 0.328	**0.504**
I-18	Do your hands swell?	0.225	− 0.115	0.126	0.180	**0.364**

Factor extraction method: principal axis factoring; Rotation method: Promax with Kaiser normalization; The bold values are the factor loadings for the items included in each factor

**Fig. 3:**
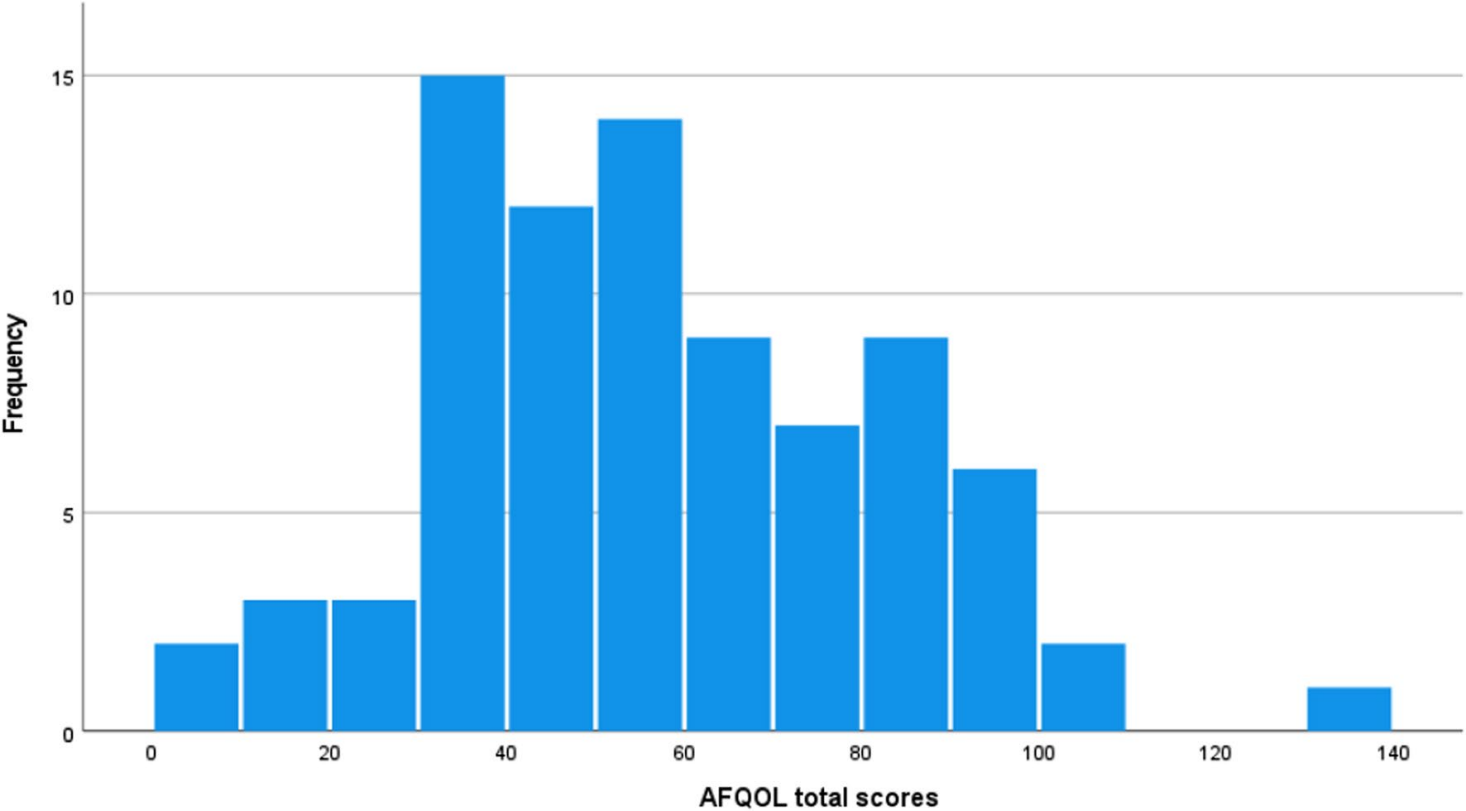
Distribution of the AFQOL total scores

Five factors were identified based on the content of the included questions. The factors were “neuropathic pain and abdominal symptoms,” “impact on work and school,” “relationship challenges,” “ophthalmologic and otolaryngologic symptoms,” and “cardiovascular and renal symptoms;” the number of items comprising each factor was 10, 6, 11, 5, and 7, respectively; and Cronbach’s alpha coefficients for the factors were 0.904, 0.875, 0.875, 0.822, and 0.834, respectively. Cronbach’s alpha for the total scale was 0.941. Descriptive statistics for each factor are shown in Table 3. The distribution of the AFQOL total scores is shown in Fig. 3, with 30–40 being the most frequent score, and the distribution having a wide base to the right. There was a significant correlation between each factor, with correlation coefficients ranging from 0.321 to 0.631 (Table 4).

**Table 3:** Description of factors of the developed scale and SF-8

	*n*	Mean	*SD*	Range
*AFQOL*				
Factor 1	83	12.7	8.37	0–34
Factor 2	83	5.7	5.53	0–21
Factor 3	83	19.7	8.30	4–42
Factor 4	83	7.4	4.43	0–20
Factor 5	83	11.4	5.56	1–25
Scale Total	83	56.8	24.81	7–137
*SF-8*				
GH	81	45.9	7.73	28.5–54.3
PF	81	45.5	8.46	19.5–54.9
RP	82	48.8	8.89	21.1–59.1
BP	82	48.6	7.79	31.4–64.5
VT	82	48.7	6.90	32.6–58.1
SF	81	46.7	9.11	28.9–55.2
MH	82	46.1	8.13	21.0–55.2
RE	82	46.9	7.66	30.1–56.7
PCS	80	46.2	7.46	29.0–57.0
MCS	80	46.4	7.79	27.2–59.2

*SD* Standard deviation; *AFQOL* Adult fabry disease quality of life scale; *SF-8* Short form-8; *GH* General health; *PF* Physical functioning; *RP* Role physical; *BP* Bodily pain; *VT* Vitality; *SF* Social functioning; *MH* Mental health; *RE* Role emotional; *PCS* Physical component summary; *MCS* Mental component summary

**Table 4:** Spearman’s correlation between AFQOL factors (*N* = 83)

	Factor 1	Factor 2	Factor 3	Factor 4	Factor 5	Scale total
Factor 1	1					
Factor 2	0.446*	1				
Factor 3	0.631*	0.432*	1			
Factor 4	0.471*	0.372*	0.326*	1		
Factor 5	0.453*	0.321*	0.506*	0.347*	1	
Scale total	0.830*	0.662*	0.832*	0.599*	0.694*	1

^*^p < 0.05; AFQOL, Adult Fabry disease quality of life scale

### Construct validity for QOL measurements

The results of the SF-8 subscales and summary scores are shown in Table 3, with all items scoring below 50. Cronbach’s alpha coefficient for SF-8 in this study was 0.959. Correlations between the factors of the newly developed scale and the SF-8 showed significant correlations for most of the factors (Table 5). Negative correlations were found for all factors of the AFQOL and all components of the SF-8. However, some non-significant correlations were also found. The correlation coefficients for the SF-8 factors “general health,” “role physical,” “social functioning,” “mental health,” “role emotional,” and “mental component summary” and Factor 2 of the AFQOL scale were not significant. The correlation coefficient for “mental component summary” and Factor 4 was also non-significant.

**Table 5:** Spearman’s correlation between the AFQOL and SF-8

SF-8	GH	PF	RP	BP	VT	SF	MH	RE	PCS	MCS
n	82	81	81	82	82	81	82	82	80	80
Factor 1	− 0.241*	− 0.350*	− 0.282*	− 0.643*	− 0.352*	− 0.346*	− 0.366*	− 0.342*	− 0.424*	− 0.295*
Factor 2	− 0.177	− 0.234*	− 0.145	− 0.241*	− 0.293*	− 0.061	− 0.11	− 0.21	− 0.245*	− 0.117
Factor 3	− 0.434*	− 0.464*	− 0.500*	− 0.569*	− 0.505*	− 0.533*	− 0.587*	− 0.638*	− 0.494*	− 0.589*
Factor 4	− 0.241*	− 0.269*	− 0.264*	− 0.483*	− 0.278*	− 0.262*	− 0.244*	− 0.245*	− 0.364*	− 0.185
Factor 5	− 0.341*	− 0.434*	− 0.384*	− 0.406*	− 0.349*	− 0.340*	− 0.325*	− 0.382*	− 0.405*	− 0.285*
Scale Total	− 0.366*	− 0.475*	− 0.436*	− 0.638*	− 0.446*	− 0.427*	− 0.456*	− 0.503*	− 0.508*	− 0.400*

^*^p < .05; *AFQOL* Adult fabry disease quality of life scale; *SF-8* Short form-8; *GH* General health; *PF* Physical functioning; *RP* Role physical; *BP* Bodily pain; *VT* Vitality; *SF* Social functioning; *MH* Mental health; *RE* Role emotional; *PCS* Physical component summary; *MCS* Mental component summary

## Discussion

### Pretest

A pretest was conducted to confirm the feasibility of using the AFQOL scale created in this study. In accordance with Devellis’s scale development guidelines, the questions were designed to avoid multiple negatives and ambiguous pronouns [19]. There were no problems with readability, and the respondents could respond using the Likert-type scale, suggesting that the questions were appropriate as scale items.

The scale also included items about parents and children from patients’ perspective. The mean age of patients with Fabry disease in Japan is late 30 s, even for the classical type, and late 40 s for the late-onset and heterozygous types [5]. It is possible that participants who had reached an advanced age had already had a parent who had passed away, making it difficult for them to answer the questions related to their parents. As the purpose of the proposed scale is to additively capture the problems that patients have in their lives, the fact that the participants did not have parents or children or were not working was not considered to negatively affect the appropriateness of the items.

### Main survey

The AFQOL developed in this study has five subscales. Three of these subscales focus on physical symptoms and two relate to employment and relationships. Refinement of the items resulted in a scale with sufficient internal consistency. Higher AFQOL scores indicate lower QOL and are negatively correlated with the SF-8. This can be considered the first results that indicate construct validity for AFQOL as a measurement of patient QOL. Contrastingly, as a newly created QOL scale, it is also important to consider discriminant validity. Therefore, it is expected that the discriminant validity of the AFQOL will be tested in the future by comparing it to measures that are not expected to correlate with QOL or that are negatively correlated. Factor 2 (“impact on work and school”) was not significantly correlated with most of the SF-8 domains. This may be related to the high number of non-responses to questions about work and school. Several items included in Factor 2 obtained a score of 0—a score given for non-response—which may have affected the correlation coefficients. Therefore, in future studies, correlations with Factor 2 for only those patients who are working should be examined, facilitating appropriate interpretations. Qualitative studies have shown that patients with rare diseases have challenges in social life, including employment [17, 23]. The AFQOL can be used to provide a detailed picture of the QOL of patients with Fabry disease. Furthermore, Factor 4 (“ophthalmologic and otolaryngologic symptoms”) was not significantly correlated with MCS. Patients with Fabry disease often experience depressive symptoms, mainly due to neuropathic pain [24]. However, few studies have determined the psychological impact of tinnitus and dizziness on patients; thus, it would be useful to examine the relationship between otolaryngological symptoms and depression using this factor included in the AFQOL to provide support to patients.

Factor 1, “neuropathic pain and abdominal symptoms,” is mainly related to symptoms that begin in childhood. The median age of neuropathic pain onset is less than 10 years old, and 58.8% of male and 40.5% of female patients presented with symptoms [7]. Similarly, gastrointestinal symptoms such as abdominal pain and diarrhea also appear during childhood [7]. Therefore, scales for pediatric patients with Fabry disease focus on neuropathic pain and abdominal symptoms [12]. However, even in adulthood, abdominal symptoms can impair patients’ QOL. Patient-reported outcome instruments that focus on gastrointestinal symptoms in adult patients have also been developed [14]. In the future, it will be important to confirm the consistency of those measures with AFQOL.

Factor 2 of the AFQOL is “impact on work and school.” To better support patients in adulthood, challenges related to employment should be considered. Previous research has suggested that the challenges in patients’ social lives are due to the impact of symptoms on their work choices and lack of understanding by supervisors [18]. ERT, one of the treatments for Fabry disease, requires hospital visits every two weeks, and patients must adjust their work and school schedules accordingly [17]. Therefore, it is important for healthcare providers, especially nurses, to show understanding for patients’ life restrictions, rather than focusing solely on symptoms. If necessary, physicians and nurses should consider providing appropriate information about the disease to those in the workplace.

Factor 3 of the AFQOL relates to “relationship challenges.” Fabry disease is difficult to diagnose because it is rare; consequently, patients are disadvantaged because of a lack of understanding of its symptoms by those around them [17]. Additionally, since Fabry disease is an X-linked disease, family members may also carry the mutation. Therefore, it is important to provide appropriate genetic counseling because a diagnosis of Fabry disease can affect family relationships and family planning [25]. Previously, heterozygous females were considered carriers; they do, however, tend to develop symptoms, which creates a gap in perception with healthcare providers [26]. In addition, the scale targeting children includes items on relationships with friends [12]. To assess the QOL of adult patients with Fabry disease, the AFQOL also covers challenges in relationships with family, friends, and healthcare providers.

Factor 4 of the AFQOL is “ophthalmologic and otolaryngologic symptoms.” Reported ophthalmologic symptoms in patients with Fabry disease include corneal verticillata, vessel tortuosity, and cataracts [27]. Patients can be affected by light glare in their daily lives [18], and 35.1% of patients with Fabry disease experience hearing loss [28]. These symptoms also affect patients’ social activities. As for neuropathic pain, the ophthalmologic and otolaryngologic symptoms are not easily noticed by others; thus, they are often unsympathetic to patients’ distress. The use of the AFQOL is also expected to help patients communicate to their healthcare providers about the restrictions in their lives owing to these symptoms.

Factor 5 of the AFQOL relates to “cardiovascular and renal symptoms.” Cardiovascular and renal symptoms are characteristic of adulthood Fabry disease [29]. Cardiovascular involvement in Fabry disease includes left ventricular hypertrophy, exertional dyspnea, and exercise angina; cardiovascular disease is the primary cause of death in patients with Fabry disease [30]. Cardiovascular symptoms are appropriate to evaluate QOL because they affect patients’ activities of daily living. The progressive accumulation of substrate also causes renal symptoms, leading to renal failure around age 50, and some patients require renal replacement therapy [31]. Qualitative studies have shown that continuous hemodialysis has a substantial impact on patients’ lives [18]. The inclusion of subscales relating to symptoms that progress in adulthood to capture the QOL of adult patients is a strength of the AFQOL.

In this study, missing values were assigned a score of 0, with the perspective that events not experienced by the patients would not be associated with decreasing QOL. However, with the exception of items regarding labor and participants’ children, missing values were seen to be missing at random; thus, handling of such data is advisable. Future studies should examine the robustness of the scale, including missing value analysis.

In this study, the content validity and factor structure of the AFQOL were determined. Future research should examine construct validity through hypothesis testing. In addition to the general QOL scale, it is important to test hypotheses according to the characteristics of the factors, such as examining the correlation between the degree of pain by brief pain inventory and Factor 1 [32].

### Clinical implications

The AFQOL is a measure that captures the overall QOL of adult patients with Fabry disease. Until now, support for patients with Fabry disease has focused solely on symptoms and treatment side effects. However, previous studies have shown that the lives of patients with Fabry disease are affected in many ways by the disease and its symptoms. Assessing the QOL of individual patients using the AFQOL will help evaluate the support that is available to them.

An objective QOL assessment with the AFQOL would allow for a detailed examination of the effectiveness of treatments such as ERT and chaperone therapy. It could be possible to observe not only the medical information of the symptoms but also the negative effects on patients’ lives from their point of view.

### Study limitations

The first limitation is the small sample size, which challenges the robustness of the analysis results. In particular, factor analysis generally requires large sample sizes. However, it has been suggested that, depending on the conditions of the analysis, a sample size of 50 cases may ensure a certain degree of robustness [33]. In addition, the low response rate of 41.5% to the questionnaire may have introduced bias in the results and interpretation. As the survey was designed to refine the scale, it is assumed that the large number of items and the lack of an honorarium affected the response rate. Future studies should accumulate results using the AFQOL in clinical practice and evaluate them with large sample sizes. In addition, when creating questionnaires, items related to work and school should include the option “not working/not in school” so that the results can be distinguished from those missing at random.

Considering the participants’ burden of answering 79 items multiple times, this study was a cross-sectional survey. Therefore, the reliability of the scale could not be adequately verified. It is preferable to use the test–retest method, in which the same participant is asked to respond twice with appropriate time periods and the results are compared according to the intraclass correlation coefficient. Moreover, the minimum important change should be verified using the anchor method or other methods to validate responsiveness. Therefore, it is necessary to acquire information from the same participant multiple times, which is a possibility in future studies if we can reduce the number of items in the scale.

The scale was also developed based on items extracted from a qualitative study of Japanese patients. Cross-cultural validation is needed. Furthermore, in creating the scale items, we could not ascertain whether important concepts from the patients’ experiences were missing. Therefore, cognitive debriefing should be conducted with patients to confirm that the AFQOL is inclusive of their experiences.

Additionally, the survey did not provide information on the disease type of the male patients, and thus did not allow for a comparison of QOL by disease type. However, it is difficult for patients to accurately determine the classic and late-onset types, which is a limitation of a patient-based questionnaire survey.

Future research should examine the discriminant and convergent validity of the AFQOL using other scales of QOL. In addition, a confirmatory factor analysis may be conducted with a larger sample to examine the validity of the factor structure of the AFQOL.

## Conclusions

Based on the interview findings of a previous study [18], a disease-specific QOL scale for adult patients with Fabry disease—the AFQOL—was developed. The AFQOL comprises 39 items and five factors; “neuropathic pain and abdominal symptoms,” “impact on work and school,” “relationship challenges,” “ophthalmologic and otolaryngologic symptoms,” and “cardiovascular and renal symptoms.” Each of the AFQOL factors was confirmed for internal consistency. The AFQOL assesses physical symptoms and social difficulties experienced by patients with Fabry disease that are not covered by general QOL scales. A particular strength of this scale is the ability to assess the impact of work and personal relationships on patients’ QOL. The AFQOL is useful in objectively assessing patients’ QOL as a group as well as allowing for consideration of support for individual patients.

## Supplementary Information

**Additional file 1: Table S1.** Scale item refinement process. **Table S2.** Distribution of responses by item in the main survey

## Data Availability

Not applicable.

## Abbreviations

AFQOL scale: Adult fabry disease quality of life scale
COSMIN: Consensus-based standards for the selection of health measurement instruments
ERT: Enzyme replacement therapy
MCS: Mental component summary
PCS: Physical component summary
QOL: Quality of life
SF-8: Short Form–8
