# Supplementary material for: Development and validation of a disease-specific quality of life scale for adult patients with Fabry disease in Japan
Source: J Patient Rep Outcomes. 2022 Nov 17;6:115. doi: 10.1186/s41687-022-00525-z (PMC9672224; doi:10.1186/s41687-022-00525-z)
Supplement: Supplementary file 1 — Additional file 1: Table S1. Scale item refinement process. Table S2. Distribution of responses by item in the main survey [file 41687_2022_525_MOESM1_ESM.docx]

**Supplementary Information**

Table S1. Scale item refinement process

| Item | Question text | Pretest | I-T  Cor | I-I  Cor | Commonality | Factor  loadings | Complete  scale |
| --- | --- | --- | --- | --- | --- | --- | --- |
| Ⅰ-1 | Do your toes begin to hurt when the ambient temperature or your body temperature rises? |  |  |  |  |  | 1 |
| Ⅰ-2 | Do your fingers begin to hurt when the ambient temperature or your body temperature rises? |  |  | ✓ |  |  |  |
| Ⅰ-3 | Do you feel pain in your elbows when the ambient temperature or your body temperature rises? |  |  | ✓ |  |  |  |
| Ⅰ-4 | Do you feel pain in your knees when the ambient temperature or your body temperature rises? |  |  |  |  |  | 1 |
| Ⅰ-5 | Are you unable to exercise due to pain? |  |  | ✓ |  |  |  |
| Ⅰ-6 | Are you unable to perform your daily activities due to pain? |  |  |  |  |  | 1 |
| Ⅰ-7 | Do your hands begin to hurt on cold days? |  |  |  |  |  | 1 |
| Ⅰ-8 | Do your feet begin to hurt on cold days? |  |  | ✓ |  |  |  |
| Ⅰ-9 | Do your hands begin to hurt while using tap water? |  |  |  |  |  | 1 |
| Ⅰ-10^a^ | Do you sweat on hot days? |  | ✓ |  |  |  |  |
| Ⅰ-11^a^ | Do you sweat while exercising? |  | ✓ |  |  |  |  |
| Ⅰ-12 | Are you immobile on hot days? |  |  |  |  |  | 1 |
| Ⅰ-13 | Do you experience any pain when your body temperature rises in the bath? |  |  |  |  |  | 1 |
| Ⅰ-14 | Do you experience palpitations? |  |  |  |  |  | 5 |
| Ⅰ-15 | Do you feel pain in your chest or difficulty breathing while walking? |  |  |  |  |  | 5 |
| Ⅰ-16 | Do you feel pain in your chest or difficulty breathing while climbing stairs? |  |  |  |  |  | 5 |
| Ⅰ-17 | Do you feel anxious that your heart symptoms will get worse? |  |  |  |  |  | 5 |
| Ⅰ-18 | Do your hands swell? |  |  |  |  |  | 5 |
| Ⅰ-19 | Do your feet swell? |  |  |  | ✓ |  |  |
| Ⅰ-20 | Do you feel anxious that your kidney symptoms will get worse? |  |  |  |  |  | 5 |
| Ⅰ-21 | Do you have trouble socializing due to Fabry disease symptoms? |  |  |  |  |  | 3 |
| Ⅰ-22 | Do you have diarrhea? |  |  |  |  |  | 1 |
| Ⅰ-23 | Do you have constipation? |  | ✓ |  |  |  |  |
| Ⅰ-24 | Do you experience abdominal pain? |  |  |  |  |  | 1 |
| Ⅰ-25 | Do you feel nauseous? |  |  |  |  |  | 1 |
| Ⅰ-26 | Do you feel that lights are too bright? |  |  |  |  |  | 4 |
| Ⅰ-27 | Do you have difficulty seeing at night? |  |  |  |  |  | 4 |
| Ⅰ-28 | Do you hear ringing in your ears? |  |  |  |  |  | 4 |
| Ⅰ-29 | Do you feel that it is difficult to have conversations because of the ringing in your ears? |  |  |  |  |  | 4 |
| Ⅰ-30 | Do you experience sudden, intense ringing in your ears? |  |  |  |  |  | 4 |
| Ⅰ-31 | Do you get dizzy? |  |  |  |  |  | 3 |
| Ⅰ-32 | Do you feel you tire easily? |  |  |  |  |  | 3 |
| Ⅰ-33 | Do you consider yourself mentally weak? |  |  |  |  |  | 3 |
| Ⅱ-1^a^ | Do you think that your treatment is controlling the pain caused by Fabry disease? |  | ✓ |  |  |  |  |
| Ⅱ-2^a^ | Do you think that your treatment is improving your Fabry disease symptoms? |  | ✓ |  |  |  |  |
| Ⅱ-3 | Do you think that your treatment is making your Fabry disease symptoms worse? |  |  |  | ✓ |  |  |
| Ⅱ-4^a^ | Are you satisfied with your current treatment? |  |  |  |  | ✓ |  |
| Ⅱ-5 | Are you anxious about the effectiveness of your current treatment? |  |  |  |  |  | 5 |
| Ⅱ-6 | Are you concerned about side effects from your current treatment? |  |  |  |  |  | 3 |
| Ⅱ-7 | Do you feel that visiting the hospital regularly for treatment influences your everyday life? |  |  |  | ✓ |  |  |
| Ⅱ-8 | Do you feel that the hospital that provides your treatment is far away? |  | ✓ |  |  |  |  |
| Ⅱ-9 | Would you like to change the medication you are currently taking for treatment? |  |  |  | ✓ |  |  |
| Ⅱ-10^a^ | Do you think that new treatments will be developed? |  | ✓ |  |  |  |  |
| Ⅲ-1 | Do you worry about your Fabry disease? |  |  |  |  |  | 3 |
| Ⅲ-2^a^ | Do you feel knowledgeable about Fabry disease? |  | ✓ |  |  |  |  |
| Ⅲ-3 | Do you feel distressed because you have been given too much information about Fabry disease? |  | ✓ |  |  |  |  |
| Ⅲ-4 | Do you feel anxious about your future because of the Fabry disease? |  |  |  |  |  | 3 |
| Ⅳ-1^a^ | Do you feel that your friends understand you? |  | ✓ |  |  |  |  |
| Ⅳ-2 | Do you feel that playing or doing activities with friends is difficult because of the Fabry disease? |  |  |  |  |  | 3 |
| Ⅳ-3^a^ | Do you think that your doctors and nurses have adequate knowledge of Fabry disease? |  | ✓ |  |  |  |  |
| Ⅳ-4^a^ | Do you think that your doctors and nurses understand the challenges that Fabry disease symptoms present in everyday life? |  | ✓ |  |  |  |  |
| Ⅳ-5 | Do you feel hurt because the people around you don’t understand you? |  |  |  |  |  | 3 |
| Ⅳ-6 | Do you feel that you can’t properly explain Fabry disease symptoms to the people around you? |  |  |  |  |  | 3 |
| Ⅳ-7^a^ | Do you feel that you can get reliable explanations about Fabry disease from your doctors or nurses? |  | ✓ |  |  |  |  |
| Ⅳ-8^a^ | Do you have opportunities to meet up with other patients with Fabry disease outside of your family? |  | ✓ |  |  |  |  |
| Ⅳ-9^a^ | Does meeting up with other patients with Fabry disease outside of your family make you feel better? |  | ✓ |  |  |  |  |
| Ⅳ-10 | Do you feel that there is insufficient education about genetic diseases in school? | ✓ |  |  |  |  |  |
| Ⅴ-1 | Do you feel that the Fabry disease symptoms impact your work or schooling? |  |  | ✓ |  |  |  |
| Ⅴ-2 | Does your Fabry disease treatment impact your work or schooling? |  |  |  |  |  | 2 |
| Ⅴ-3 | Do you feel that working outside is difficult? |  |  |  |  |  | 2 |
| Ⅴ-4 | Do you feel that your hearing difficulty affects work or schooling? |  |  |  |  |  | 2 |
| Ⅴ-5^a^ | Do you think that your bosses and coworkers (teachers and classmates) have adequate knowledge of Fabry disease? |  | ✓ |  |  |  |  |
| Ⅴ-6^a^ | Do you think that your bosses and coworkers (teachers and classmates) understand the challenges you face at work due to Fabry disease symptoms? |  | ✓ |  |  |  |  |
| Ⅴ-7 | Do you get scolded by those around you because you become irritable due to pain from Fabry disease? |  |  |  |  |  | 2 |
| Ⅴ-8 | Do you feel bitter because of prejudice toward Fabry disease at your workplace or school? |  |  |  |  |  | 2 |
| Ⅴ-9 | Do people at your workplace or school ever make snide remarks about your Fabry disease symptoms? |  |  |  |  |  | 2 |
| Ⅵ-1^a^ | Do you talk with your family about Fabry disease? |  | ✓ |  |  |  |  |
| Ⅵ-2^a^ | Do you think you understand your family members’ symptoms? |  | ✓ |  |  |  |  |
| Ⅵ-3^a^ | Do your parents explain things about Fabry disease to you? |  | ✓ |  |  |  |  |
| Ⅵ-4^a^ | Do you feel that your parents understand your symptoms? |  | ✓ |  |  |  |  |
| Ⅵ-5^a^ | Do you talk with relatives outside your immediate family about Fabry disease? |  | ✓ |  |  |  |  |
| Ⅵ-6 | Do you have trouble socializing with relatives due to the Fabry disease? |  |  |  |  |  | 3 |
| Ⅵ-7 | Do you think it is your parents’ fault that you have Fabry disease? |  | ✓ |  |  |  |  |
| Ⅵ-8^a^ | Do you explain things about Fabry disease to your child(ren)? |  | ✓ |  |  |  |  |
| Ⅵ-9 | Do you feel sorry that your child(ren) is/are suffering from the symptoms of Fabry disease? |  | ✓ |  |  |  |  |
| Ⅵ-10 | Do you feel sorry that your child(ren) is/are suffering because they have Fabry disease? |  | ✓ |  |  |  |  |
| Ⅵ-11 | Do you feel it is your fault that your child(ren) has/have Fabry disease? |  | ✓ |  |  |  |  |
| Ⅶ-1 | Do you feel that your Fabry disease symptoms impact getting married? |  | ✓ |  |  |  |  |
| Ⅶ-2 | Do you feel that being diagnosed with Fabry disease impacts getting married? |  | ✓ |  |  |  |  |
| Ⅶ-3 | Do you feel that it is difficult to have children due to the Fabry disease? |  | ✓ |  |  |  |  |

The ✓ is in the stage where the scale item was dropped out of the refinement process. Items included in the completed scale are listed with factor numbers; ^a^Reverse scoring items, I-T Cor: Item-total correlation, I-I Cor: Item-item correlation

Table S2. Distribution of responses by item in the main survey (%) (*N* = 83)

| Item | Always | Often | Sometimes | Seldom | Never | No answer | No child |
| --- | --- | --- | --- | --- | --- | --- | --- |
| Ⅰ-1 | 10.8 | 12.0 | 25.3 | 20.5 | 30.1 | 1.2 | - |
| Ⅰ-2 | 9.6 | 8.4 | 28.9 | 19.3 | 32.5 | 1.2 | - |
| Ⅰ-3 | 1.2 | 6.0 | 4.8 | 21.7 | 65.1 | 1.2 | - |
| Ⅰ-4 | 1.2 | 4.8 | 12.0 | 24.1 | 56.6 | 1.2 | - |
| Ⅰ-5 | 6.0 | 8.4 | 18.1 | 22.9 | 43.4 | 1.2 | - |
| Ⅰ-6 | 2.4 | 7.2 | 10.8 | 34.9 | 43.4 | 1.2 | - |
| Ⅰ-7 | 4.8 | 8.4 | 19.3 | 27.7 | 36.1 | 3.6 | - |
| Ⅰ-8 | 2.4 | 13.3 | 19.3 | 24.1 | 37.3 | 3.6 | - |
| Ⅰ-9 | 3.6 | 7.2 | 9.6 | 27.7 | 48.2 | 3.6 | - |
| Ⅰ-10^a^ | 27.7 | 20.5 | 13.3 | 27.7 | 8.4 | 2.4 | - |
| Ⅰ-11^a^ | 24.1 | 20.5 | 21.7 | 22.9 | 8.4 | 2.4 | - |
| Ⅰ-12 | 12.0 | 7.2 | 25.3 | 32.5 | 21.7 | 1.2 | - |
| Ⅰ-13 | 10.8 | 10.8 | 20.5 | 25.3 | 27.7 | 4.8 | - |
| Ⅰ-14 | 4.8 | 14.5 | 38.6 | 26.5 | 15.7 | 0.0 | - |
| Ⅰ-15 | 2.4 | 13.3 | 30.1 | 39.8 | 14.5 | 0.0 | - |
| Ⅰ-16 | 9.6 | 14.5 | 31.3 | 22.9 | 19.3 | 2.4 | - |
| Ⅰ-17 | 14.5 | 13.3 | 36.1 | 26.5 | 9.6 | 0.0 | - |
| Ⅰ-18 | 1.2 | 16.9 | 15.7 | 34.9 | 30.1 | 1.2 | - |
| Ⅰ-19 | 7.2 | 24.1 | 25.3 | 24.1 | 19.3 | 0.0 | - |
| Ⅰ-20 | 12.0 | 10.8 | 33.7 | 30.1 | 13.3 | 0.0 | - |
| Ⅰ-21 | 16.9 | 15.7 | 27.7 | 20.5 | 16.9 | 2.4 | - |
| Ⅰ-22 | 10.8 | 22.9 | 30.1 | 22.9 | 10.8 | 2.4 | - |
| Ⅰ-23 | 4.8 | 13.3 | 30.1 | 39.8 | 12.0 | 0.0 | - |
| Ⅰ-24 | 4.8 | 18.1 | 31.3 | 33.7 | 10.8 | 1.2 | - |
| Ⅰ-25 | 1.2 | 4.8 | 20.5 | 44.6 | 27.7 | 1.2 | - |
| Ⅰ-26 | 3.6 | 12.0 | 34.9 | 25.3 | 22.9 | 1.2 | - |
| Ⅰ-27 | 4.8 | 20.5 | 24.1 | 31.3 | 19.3 | 0.0 | - |
| Ⅰ-28 | 20.5 | 19.3 | 24.1 | 20.5 | 15.7 | 0.0 | - |
| Ⅰ-29 | 6.0 | 9.6 | 10.8 | 43.4 | 30.1 | 0.0 | - |
| Ⅰ-30 | 2.4 | 4.8 | 24.1 | 33.7 | 34.9 | 0.0 | - |
| Ⅰ-31 | 2.4 | 6.0 | 34.9 | 37.3 | 19.3 | 0.0 | - |
| Ⅰ-32 | 25.3 | 32.5 | 22.9 | 12.0 | 7.2 | 0.0 | - |
| Ⅰ-33 | 16.9 | 16.9 | 26.5 | 28.9 | 10.8 | 0.0 | - |
| Ⅱ-1^a^ | 7.2 | 10.8 | 19.3 | 36.1 | 20.5 | 6.0 | - |
| Ⅱ-2^a^ | 6.0 | 14.5 | 12.0 | 48.2 | 15.7 | 3.6 | - |
| Ⅱ-3 | 0.0 | 3.6 | 3.6 | 31.3 | 59.0 | 2.4 | - |
| Ⅱ-4^a^ | 16.9 | 21.7 | 36.1 | 18.1 | 2.4 | 4.8 | - |
| Ⅱ-5 | 8.4 | 7.2 | 36.1 | 33.7 | 10.8 | 3.6 | - |
| Ⅱ-6 | 3.6 | 3.6 | 13.3 | 55.4 | 20.5 | 3.6 | - |
| Ⅱ-7 | 20.5 | 21.7 | 31.3 | 15.7 | 7.2 | 3.6 | - |
| Ⅱ-8 | 14.5 | 14.5 | 25.3 | 31.3 | 10.8 | 3.6 | - |
| Ⅱ-9 | 2.4 | 6.0 | 21.7 | 43.4 | 22.9 | 3.6 | - |
| Ⅱ-10^a^ | 27.7 | 28.9 | 27.7 | 9.6 | 2.4 | 3.6 | - |
| Ⅲ-1 | 22.9 | 16.9 | 27.7 | 28.9 | 2.4 | 1.2 | - |
| Ⅲ-2^a^ | 1.2 | 25.3 | 45.8 | 25.3 | 0.0 | 2.4 | - |
| Ⅲ-3 | 0.0 | 3.6 | 19.3 | 48.2 | 26.5 | 2.4 | - |
| Ⅲ-4 | 27.7 | 13.3 | 41.0 | 15.7 | 1.2 | 1.2 | - |
| Ⅳ-1^a^ | 7.2 | 24.1 | 20.5 | 31.3 | 14.5 | 2.4 | - |
| Ⅳ-2 | 8.4 | 10.8 | 33.7 | 27.7 | 18.1 | 1.2 | - |
| Ⅳ-3^a^ | 15.7 | 30.1 | 21.7 | 30.1 | 2.4 | 0.0 | - |
| Ⅳ-4^a^ | 13.3 | 22.9 | 31.3 | 30.1 | 1.2 | 1.2 | - |
| Ⅳ-5 | 6.0 | 13.3 | 22.9 | 44.6 | 13.3 | 0.0 | - |
| Ⅳ-6 | 10.8 | 31.3 | 22.9 | 25.3 | 8.4 | 1.2 | - |
| Ⅳ-7^a^ | 16.9 | 25.3 | 25.3 | 30.1 | 2.4 | 0.0 | - |
| Ⅳ-8^a^ | 7.2 | 8.4 | 25.3 | 36.1 | 21.7 | 1.2 | - |
| Ⅳ-9^a^ | 4.8 | 16.9 | 30.1 | 30.1 | 18.1 | 0.0 | - |
| Ⅴ-1 | 13.3 | 14.5 | 13.3 | 21.7 | 6.0 | 31.3 | - |
| Ⅴ-2 | 14.5 | 14.5 | 15.7 | 20.5 | 3.6 | 31.3 | - |
| Ⅴ-3 | 16.9 | 13.3 | 12.0 | 18.1 | 7.2 | 32.5 | - |
| Ⅴ-4 | 8.4 | 13.3 | 14.5 | 19.3 | 14.5 | 30.1 | - |
| Ⅴ-5^a^ | 0.0 | 1.2 | 3.6 | 20.5 | 43.4 | 31.3 | - |
| Ⅴ-6^a^ | 0.0 | 4.8 | 13.3 | 22.9 | 27.7 | 31.3 | - |
| Ⅴ-7 | 0.0 | 1.2 | 4.8 | 25.3 | 38.6 | 30.1 | - |
| Ⅴ-8 | 1.2 | 4.8 | 7.2 | 25.3 | 30.1 | 31.3 | - |
| Ⅴ-9 | 1.2 | 2.4 | 7.2 | 19.3 | 38.6 | 31.3 | - |
| Ⅵ-1^a^ | 9.6 | 9.6 | 36.1 | 41.0 | 2.4 | 1.2 | - |
| Ⅵ-2^a^ | 12.0 | 30.1 | 34.9 | 10.8 | 3.6 | 8.4 | - |
| Ⅵ-3^a^ | 2.4 | 8.4 | 10.8 | 26.5 | 43.4 | 8.4 | - |
| Ⅵ-4^a^ | 15.7 | 12.0 | 21.7 | 13.3 | 24.1 | 13.3 | - |
| Ⅵ-5^a^ | 0.0 | 2.4 | 14.5 | 39.8 | 41.0 | 2.4 | - |
| Ⅵ-6 | 2.4 | 2.4 | 12.0 | 38.6 | 42.2 | 2.4 | - |
| Ⅵ-7 | 2.4 | 3.6 | 16.9 | 22.9 | 50.6 | 3.6 | - |
| Ⅵ-8^a^ | 4.8 | 6.0 | 31.3 | 20.5 | 4.8 | 3.6 | 28.9 |
| Ⅵ-9 | 24.1 | 10.8 | 6.0 | 13.3 | 4.8 | 10.8 | 30.1 |
| Ⅵ-10 | 24.1 | 8.4 | 7.2 | 13.3 | 7.2 | 9.6 | 30.1 |
| Ⅵ-11 | 21.7 | 10.8 | 3.6 | 13.3 | 10.8 | 9.6 | 30.1 |
| Ⅶ-1 | 33.7 | 24.1 | 22.9 | 12.0 | 4.8 | 2.4 | - |
| Ⅶ-2 | 31.3 | 26.5 | 25.3 | 9.6 | 4.8 | 2.4 | - |
| Ⅶ-3 | 27.7 | 24.1 | 21.7 | 15.7 | 7.2 | 3.6 | - |

See Table S1 for item text; ^a^Reverse scoring items
